# The high-osmolarity glycerol (HOG) pathway in *Candida auris*

**DOI:** 10.1128/mbio.03538-24

**Published:** 2025-01-29

**Authors:** Hajar Yaakoub, Vincent Courdavault, Nicolas Papon

**Affiliations:** 1 University of Angers, Brest University, IRF, SFR ICAT, Angers, France; 2 Nantes-Université, INRAE, UMR 1280, PhAN, Nantes, France; 3 Biomolécules et Biotechnologies Végétales, Université de Tours, Tours, France; CDC, Georgia, Atlanta, USA

**Keywords:** *Candida auris*, HOG, signaling, cell wall, virulence

## Abstract

The emerging fungal pathogen *Candida auris* is known for its strong skin tropism and resilience against antifungal and disinfection treatment, posing a significant challenge for healthcare units. Although efforts to identify the effectors of its unique pathogenic behavior have been insightful, the role of the high-osmolarity glycerol (HOG) pathway in this context remains unexplored. The study by Shivarathri and co-workers (R. Shivarathri, M. Chauhan, A. Datta, D. Das et al., mBio 15:e02748-24, 2024, https://doi.org/10.1128/mbio.02748-24) sought to address this gap. This report indeed advances our understanding of the critical role of the HOG pathway in *C. auris* pathogenicity by emphasizing its involvement in skin colonization, biofilm formation, and evasion of phagocyte attack.

## COMMENTARY

Nearly a quarter of annual invasive fungal infections are caused by members of the *Candida* genus (1). Within this group, the CTG clade includes several major pathogenic species, such as *Candida albicans*, *Candida tropicalis*, and *Candida parapsilosis*, alongside the notably divergent *C. auris* (2). Unlike the other species, *C. auris* has been voiced as “super fungus,” to highlight its significant menace to public health. Its first isolation in Japan in 2009 from an otomycosis case was relatively inauspicious (3), as it has since taken just one decade for this fungus to transition from an unidentified *Candida* species to a globally recognized health threat by the World Health Organization and the Centers for Disease Control and Prevention. This concern stems from the dramatic emergence of drug-resistant and skin-tropic *C. auris* in healthcare facilities. Rapid inter- and intra-hospital transmission, coupled with its surface-lingering ability and diagnostic challenges, has driven clonally disseminated outbreaks, thus leading to high mortality rates, particularly among critically ill patients (4–9). Genomic surveillance of emerging strains has identified six distinct genetic lineages, primarily clustered into six geographic clades and believed to have emerged concurrently (10–13), with further genotypic variants expected to arise. This rapid spread of *C. auris* is not fully understood, but several factors have been speculated. This includes not only the climate changes that may have favored the fungus spread due to its thermotolerant nature (14, 15) but also the widespread use of antifungals in clinics and agriculture that could have led to the emergence of resistant strains (16–18). Genetic recombination and host-induced *de novo* mutations have been proposed as contributors to genetic diversity (2, 19). The resulting inter-clade and intra-clade variability has been shown to culminate in phenotypic changes including antifungal resistance, outbreak propensity, and pathogenicity (20). Nonetheless, considerable gaps remain in understanding the epigenetic mechanisms that underpin the environmental adaptability and pathogenicity in *C. auris*. Therefore, studying how it evolved into a resilient pathogen is vital for developing effective prevention and control strategies.

**Fig 1:**
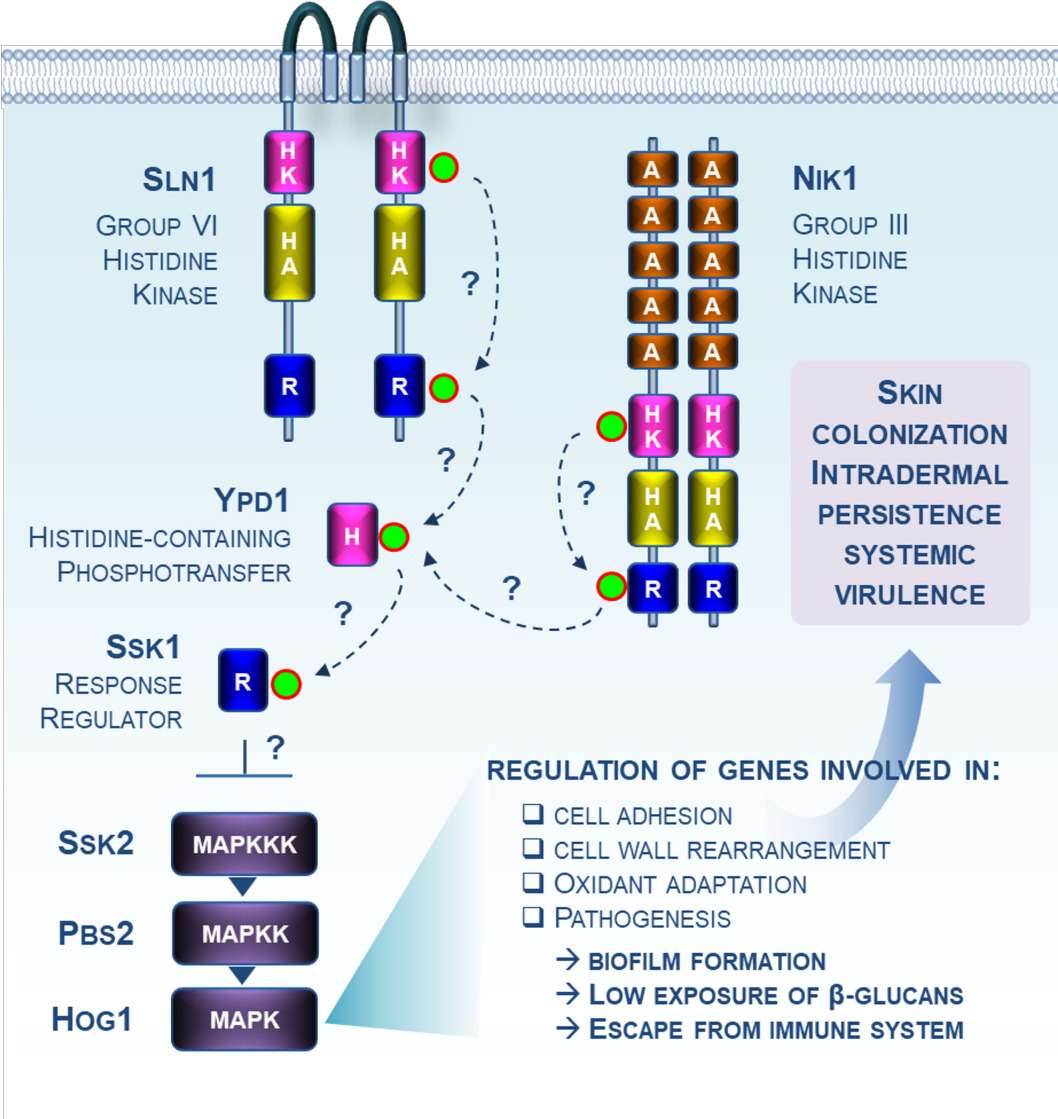
MAP kinase Hog1 governs skin colonization and intradermal persistence in *C. auris*. The *C. auris* genome encodes a group VI histidine kinase (Sln1), a group III histidine kinase (Nik1), a histidine-containing phosphotransfer protein (Ypd1), a response regulator (Ssk1), and three elements of the canonical high-osmolarity glycerol (HOG) mitogen-activated protein kinase (MAPK) pathway (Ssk2, Pbs2, and Hog1). All these components are potentially part of the so-called two-component signaling system (TCS) in *C. auris*. The communication between these proteins by phosphotransfer (green circles and arrows) is still unknown. In the present report, Shivarathri and colleagues demonstrated that Hog1 is required for efficient skin colonization, intradermal persistence, and systemic virulence. Authors notably highlighted that Hog1 is involved in the regulation of genes associated with processes such as cell adhesion, cell wall rearrangement, and pathogenesis. Consequently, they revealed the prominent role of Hog1 in maintaining cell wall architecture (regulation of cell-surface β-glucan exposure and chitin content), biofilm formation, and fungal survival when challenged with immune cells. Abbreviations: HK, histidine kinase domain; HA, H-ATPase domain; R, receiver domain; H, histidine-containing phosphotransfer domain; A, histidine kinase, adenyl cyclase, methyl-accepting protein, and phosphatase domain; MAPK, mitogen-activated protein kinase; MAPKK, MAPK kinase MAPK, MAPKKK, MAPKK kinase.

Skin colonization by *C. auris* now represents a major risk factor for candidemia (21), especially if invasive procedures take place (22, 23). This remarkable ability to adhere to both human skin and inert surfaces is key to its persistence and nosocomial transmission (21). The genetic basis of this adhesion and biofilm formation has been recently investigated, identifying key regulators such as the Ume6 transcription factor (24) and genes encoding agglutinin-like sequences Als4122, adhesin Iff4109, and the surface colonization factor Scf1 (20, 25). These adhesion elements have also been shown to contribute to the fungus’s phenotypic plasticity (ability to form aggregates and pseudohyphae), which in turn enhances its invasiveness and allows escape from immune attack (20, 26, 27). This is not surprising, given the importance of the cell wall components in host-pathogen interactions. Persistence of *C. auris* in hospital environments has also prompted interest in studying its ability to withstand stress, a common characteristic among virulent pathogens. This has drawn attention to the high-osmolarity glycerol (HOG) mitogen-activated protein kinase (MAPK) pathway, which is a key molecular apparatus underpinning fungal adaptation to stress (28, 29). When triggered by stresses, the HOG pathway undergoes a series of phosphorylation events involving three components: MAP kinase kinase kinase (MAP-KKK), MAP kinase kinase (MAPKK), and Hog1 MAP kinase. When activated on its TGY (threonine-glycine-tyrosine) motif, Hog1 coordinates transcriptional and post-transcriptional cellular reprogramming. The transmission of signals to the HOG pathway is primarily facilitated by a two-component system (TCS), a multi-step phosphorelay process involving histidine kinase sensors (HK) and a histidine-containing phosphotransfer protein (HPT), which mediates the interaction between the HK and a response regulator (RR) (Fig. 1) (28). Despite its relatively small number of components, the HOG pathway serves as a crucial regulatory hub, orchestrating specific responses to a variety of abiotic and biotic stresses. Additionally, the pathway has been reported to play critical roles in regulating cell wall integrity, immune interactions, and virulence in many fungal species (28, 29). In this respect, Shivarathri et al. (30) considered the HOG pathway as an alluring candidate for further investigation into *C. auris* pathogenicity. Until now, the HOG pathway had already been investigated in *C. auris* by two studies (31, 32), which elucidated the role of Hog1 in cellular morphology, cell wall regulation, stress resistance, and resistance to both amphotericin B and caspofungin. Day et al. (32) also demonstrated its role in virulence in a *Caenorhabditis elegans* model. However, transcriptomic analysis had not been applied, and the contribution of Hog1 to skin colonization and virulence in a murine model had not been determined. In a new report published in *mBio*, Shivarathri et al. (30) addressed this gap by characterizing a *C. auris hog1*Δ mutant using RNA sequencing (RNA-seq) technique, *ex vivo* survival assays, and *in vivo* murine skin and systemic models of infection. The authors substantiated, for the first time, the involvement of Hog1 in three key pathogenic mechanisms: (i) regulation of adhesion and biofilm formation, (ii) β-glucan masking and resistance to phagocyte killing, and (iii) most importantly, skin colonization and virulence (Fig. 1).

First and importantly, Shivarathri and colleagues demonstrated that *HOG1* deletion led to a drastic regulation of roughly 40% of *C. auris* genome, accounting for downregulation or upregulation of a total of 2,060 genes (30). Although many of these genes remain uncharacterized, a significant portion are predicted to function in adhesion and biofilm formation, cell wall architecture, and fungus-host interaction. Genes from the first category included several ones with altered expression, such as *WOR1* (white-opaque regulator 1), *SAP9* (secreted aspartyl protease), and *IFF4109*. In contrast, *ALS4112* (designated *ALS5*) was upregulated. Interestingly, the expression of the prominent surface colonization factor *SCF1* remained unchanged. Still, these transcriptional changes resulted in significant alterations in biofilm structure. Compared with wild-type, the *hog1*Δ mutant exhibited a weak biofilm with reduced volume, a discontinuous growth pattern, and sparse multicellular clusters. The fact that the expression of *ALS4112*, encoding the adhesin critical to adhesion and biofilm formation in *C. auris*, was induced in the mutant with impaired biofilm formation suggests the activation of a compensatory pathway, likely involving the Cek1 (cell wall integrity pathway). This hypothesis is supported by evidence that *HOG1* deletion in *C. auris* induces basal phosphorylation of Cek1 (31). Inklings from fungal studies also suggest the rewiring of signaling pathways in favor of biofilm maturation (33). It is worth mentioning that the larger cell phenotype of the *hog1*Δ mutant, as observed in a previous study using the same mutant strain (31), may also contribute to impaired biofilm formation. Together, these findings highlighted Hog1 as a potential target for antifungal drug development, especially for tackling biofilm-forming and adherent pathogens such as *C. auris*.

Enrichment of genes involved in cell wall organization was observed in the downregulated gene set in the *hog1*Δ mutant, including those encoding chitin synthase (*CHS2*) and β-glucanases (*BGL2* and *XOG1*) (30). This may have led to an increase in cell wall β-glucan content and a decrease in chitin levels. Consequently, the mutant was more easily recognizable by innate immune cells, triggering an elevated H_2_O_2_ production by neutrophils. The mutant was also more susceptible to phagocytic killing, likely due to compromised antioxidant defenses and impaired vacuolar transport. For instance, several downregulated genes in the *hog1*Δ mutant encode key antioxidant enzymes, including superoxide dismutase, thioredoxin, and flavohemoglobin, all of which are crucial for fungi to militate oxidative and nitrosative stress (28, 29). Although these findings underscore the role of Hog1 in maintaining the constitutive expression of antioxidant genes, complementary transcriptomic analysis performed under stressed conditions would provide a clearer glimpse into Hog1-mediated antioxidant gene induction in response to oxidative killing.

With conclusive evidence substantiating the role of Hog1 in cell wall architecture, biofilm formation, and defense against phagocyte-mediated killing, the authors next probed the capacity of the *hog1*Δ mutant to colonize the skin and persist intradermally (30). They found a marked reduction in fungal burden in mice infected with the mutant via both epicutaneous and intradermal routes, which was not due to an increase in myeloid phagocyte infiltration. Moreover, the mutant resulted in lower fungal loads in the kidneys and brain. These findings provide insights into the role of the HOG pathway in masking β-glucan, rendering the fungus unrecognizable to the immune system and potentially aiding its dissemination. In this regard, it may be valuable to compare the *hog1*Δ mutant across different genetic backgrounds to determine if this regulation is strain-dependent.

In summary, the study by Shivarathri et al. (30) extends the breadth of roles already ascribed to the HOG pathway in *C. auris* pathogenesis, particularly in skin colonization, biofilm formation, and resistance to immune defenses. This highlights the therapeutic promise of Hog1, as its high conservation across fungi positions it as an appealing target for broad-spectrum antifungals, potentially overcoming issues linked to *C. auris* genetic variability. Additionally, exploring the role of the HOG pathway in *C. auris* epigenetic regulation may reveal mechanisms that underlie its shift from commensalism to virulence, thus enabling treatments that specifically impair pathogenicity without disrupting host microbiota. Although new cell-wall-targeting antifungals like fosmanogepix and ibrexafungerp show potential activities, lessons learned from past cell-wall therapies underscore the importance of targeting deeper, conserved pathways like the HOG pathway to achieve lasting efficacy against pan-resistant *C. auris*.

From a fundamental perspective, it will be eventually relevant to characterize the upstream TCS governing the *C. auris* HOG pathway, particularly the role of HKs which are known to act as primary sensors in these fungal cell signaling pathways (Fig. 1) (34). Indeed, although *C. albicans* and other clinically important *Candida* species display three structurally distant HKs including Sln1 (group VI HK), Nik1 (group III HK), and Chk1 (group X HK), *C. auris* stands out with the presence of only Sln1 and Nik1 orthologs in its genome (35). Since the major role of Chk1 in biofilm formation, morphogenetic transition, and virulence has been well established in *C. albicans*, there is considerable excitement in deciphering how *C. auris* has rewired their TCS components without a Chk1 sensor for stress adaptation and pathogenicity.
